# Analysis of biophysical and functional consequences of tropomyosin–fluorescent protein fusions

**DOI:** 10.1002/1873-3468.12346

**Published:** 2016-08-23

**Authors:** Holly R. Brooker, Michael A. Geeves, Daniel P. Mulvihill

**Affiliations:** ^1^ School of BiosciencesUniversity of KentCanterburyUK

**Keywords:** acetylation, actin cytoskeleton, Cdc8, fission yeast, *Schizosaccharomyces pombe*

## Abstract

The dynamic nature of actin polymers is modulated to facilitate a diverse range of cellular processes. These dynamic properties are determined by different isoforms of tropomyosin which are recruited to distinct subpopulations of actin polymers to differentially regulate their functional properties. This makes tropomyosin an attractive target for labelling discrete actin populations. We have assessed the effect of different fluorescent labelling strategies for this protein. Although tropomyosin–fluorescent fusions decorate actin *in vivo*, they are either nonfunctional or perturb regulation of actin nucleation and cell cycle timings. Thus, conclusions and physiological relevance should be carefully evaluated when using tropomyosin fusions.

## Abbreviations

**CAR**, contractile actomyosin ring

**FP**, fluorescent proteins

**Tpm**, tropomyosins

Many dynamic processes within the eukaryotes are driven by the actin cytoskeleton. Actin polymers can generate force to remodel membrane architecture as well as act as tracks for molecular motors. It is the dynamic nature of the actin polymers that is critical for their cellular function. These key properties are regulated by diverse actin‐binding proteins, which drive nucleation, stabilisation, cross‐linking or even depolymerisation of actin complexes 1.

One such class of actin‐binding proteins are the tropomyosins (Tpm), a highly conserved class of dimeric α‐helical coiled‐coil proteins which associate with and stabilise actin 2. Tpm dimers interact in an end‐to‐end fashion to generate filaments that associate with and coil around the F‐actin polymer. These Tpm cofilaments play a central role in stabilising actin filament dynamics in muscle cells and therefore regulate muscle contraction 3. In nonmuscle cells, Tpm plays a critical role in stabilising and also defining the biophysical properties of a specific actin polymer as well as modulating its ability to interact with other proteins and thereby regulate its function 4.

Yeast and animal cells express multiple isoforms of Tpm, each of which associate with specific actin structures at a specific place and time, in an exquisitely orchestrated manner, to fine tune the biophysical nature of each actin polymer in a specific way 2. This makes the different Tpms attractive targets as markers during the analysis of specific actin structures and functions. There is sequence diversity along the length of the different Tpm isoforms, but it is the ends of the protein that seem to be key in defining their function. The termini of the Tpm dimer not only define the structural and charge landscape at the end of the actin‐Tpm copolymer but also determine the nature of the end to end contacts between adjacent dimers. This overlap region can vary significantly between different Tpm isoforms and is essential for defining the stability of the Tpm polymer and its ability to bind cooperatively to F‐actin. Indeed, seemingly innocuous modifications to the charge on the polypeptide in these overlapping regions can have a profound effect upon the stability of the actin‐Tpm complex. For example, in many cell types the addition of acetyl group to the amino‐terminal residue of the Tpm stabilises the alpha‐helical structure of each coiled‐coil structure to facilitate its interaction with carboxyl region of adjacent Tpm molecules 5, 6. Thus, small changes in the charge and shape of the Tpm protein have a dramatic impact upon protein interactions and function.

The molecular‐genetic plasticity of yeast model systems provides an excellent setting for studying the impact protein modifications have upon its cellular structure. Yeasts express a smaller number of Tpm isoforms in comparison to metazoan cells, which makes them an attractive organism in which to characterise specific Tpm functions. The fission yeast *Schizosaccharomyces pombe* has a single tropomyosin, Tpm^Cdc8^, which exists in amino‐terminally acetylated and nonacetylated forms within the cell 7, 8. Depending upon its acetylation status, Tpm^Cdc8^ associates with the actomyosin ring during mitosis to facilitate myosin II motor activity and cytokinesis, or with cytoplasmic actin filaments upon which myosin V motors move 9, 10, 11. It thus provides a simple system for studying the regulation of function of this essential cytoskeletal protein.

Fluorescent Proteins (FP) provide an attractive method for following protein dynamics within a live cell 12. FPs have been developed with a variety of spectral properties to facilitate diverse functional analysis. However, FP fusions can impact protein function by inhibiting normal protein folding or affecting interactions with other proteins. Not surprisingly both microtubule and actin cytoskeletons are acutely sensitive to fluorescent labelling of protein components 13. Diverse FP‐labelled markers exist to follow the dynamics of the actin cytoskeleton in a live cell context, however, many have subsequently been shown to alter the behaviour and organisation of the polymers within cells 14, 15, 16. Tpms are attractive candidates for markers of specific actin structures and have been used to follow filament dynamics in diverse cell types, in a live cell context 17, 18, 19, 20, 21. However, it is unclear how fusing a FP to the termini of the Tpm protein impacts its normal function.

Here, we describe *in vitro* and *in vivo* analyses of amino‐ and carboxyl‐terminal fusions between the fission yeast tropomyosin, Tpm^Cdc8^, and a monomeric fluorescent protein. We establish that while Tpm^Cdc8^‐carboxyl terminal fusions disrupted the ability of the protein to polymerise or associate with actin *in vitro,* the amino‐terminal fusion formed filaments and associated with actin in a manner similar to amino‐terminally acetylated endogenous Tpm^Cdc8^. However, while this protein facilitated the formation of a functional contractile actomyosin ring (CAR) and cell growth in cells lacking functional endogenous Tpm^Cdc8^, it disrupted the normal timing of cell division and normal myosin V movements. Thus, each Tpm‐FP fusion has a significant impact upon the function of this critical cytoskeletal protein.

## Materials and methods

### Molecular biology

pJC20*cdc8* ^*+*^ and pREP41*cdc8* ^*+*^ were described previously 8. *cdc8‐Gly*_*3*_ *‐Cerulean3‐His*_*6*_ *and His*_*6*_ *‐Cerulean3‐Gly*_*3*_ *‐cdc8* terminal fusions were synthesised as Nde1‐BamH1 fragments (Thermo Fisher Scientific, Waltham, MA, USA) and cloned into pJC20 22 and pREP41 23 bacterial and fission yeast expression vectors.

### Cell culture

The yeast strains used in the study were h^−^ *leu1.32*; h^−^ *cdc8.110 myo2.mCherry leu1.32* and h^−^ *cdc8.110 myo52.mNeongreen leu1.32*. Cell culture and maintenance were carried out according to 24 using Edinburgh minimal medium with Glutamic acid nitrogen source (EMMG). Growth rates were determined from growth curves generated using a BMG Spectrostar Nano plate reader while shaking cells at 36 °C. All cells were maintained as early to midlog phase cultures for 48 h before being used for all analyses.

### Microscopy

Imaging was undertaken as described previously 9. Timelapse images of > 10 cells undergoing cytokinesis were used to calculate each CAR constriction rate.

### Protein purification

Unlabelled Tpm^Cdc8^ protein was purified as described previously 8. Tpm^Cdc8^ Cerulean3 fusions were expressed from pJC20 *Tpm* ^*cdc8*^ *‐Cerulean3‐His*_*6*_ and pJC20*His*_*6*_ *‐Cerulean3‐Tpm* ^*cdc8*^ in either BL21 DE3 or BL21 DE3 pNatB 25 cells. Midlog cultures were grown for 3 h with 100 mg·L^−1^ IPTG. Cells were harvested, resuspended in 30 mL lysis buffer (20 mm Tris pH 7.5, 100 mm NaCl, 2 mm EGTA and 5 mm MgCl_2_), lysed by sonication. Debris and insoluble components were removed by centrifugation and the resulting supernatant was incubated with 10 mg·L^−1^ DNase and 10 mg·L^−1^ RNase at 4 °C for 1 h before isolating His_6_‐labelled fusions on nickel‐agarose columns and eluting with imidazole. After buffer exchange into FPLC loading buffer (5 mm Tris pH 7.0, 100 mm NaCl) the FP‐labelled Tpm^Cdc8^ was further purified with FPLC using 2 × 5 mL Pharmacia HiTrap‐Q columns in tandem, by elution with a 100–900 mm NaCl gradient. Fusions were isolated from appropriate fractions, and concentrated in 5 mm Tris pH 7.0. The purity and mass of the proteins were determined by mass spectroscopy, while parallel Bradford, gel densitometry and spectroscopic analyses were used in parallel to confirm protein concentrations. Rabbit actin was purified as described previously 26.

### Circular dichroism

Measurements were made in 1‐mm quartz cuvettes using a Jasco 715 spectropolarimeter. Cdc8 proteins were diluted in CD buffer (10 mm Potassium phosphate, 500 mm NaCl, 5 mm MgCl_2_ pH 7.0) to a concentration of 0.4 mg·mL^−1^. Thermal unfolding data were obtained by monitoring the CD signal at 222 nm with a heating rate of 1 °C·min^−1^. At completion of the melting‐curve the sample was cooled at a rate of 20 °C·min^−1^. CD data are presented as differential absorption (∆A).

### Viscomentry

A Cannon‐Manning semimicroviscometer was used to determine the viscosity of 20 μm Cdc8 samples at 20 ± 1 °C in 1 mL of viscometry buffer (20 mm MOPS, 5 mm MgCl_2_ pH 7.0). NaCl concentration was increased from 0 to 250 mm. Kinematic viscosity was calculated using the manufacturer's predetermined microviscometer kinematic viscosity constant (0.03235) and the average efflux time (typically 30–40 s), calculated from five observations per sample at each NaCl concentration.

### Actin‐binding assay

Cosedimentation assays were performed at 25 °C by mixing 10 μm actin with increasing concentrations of Tpm as described previously 27.

## Results

**Figure 1:**
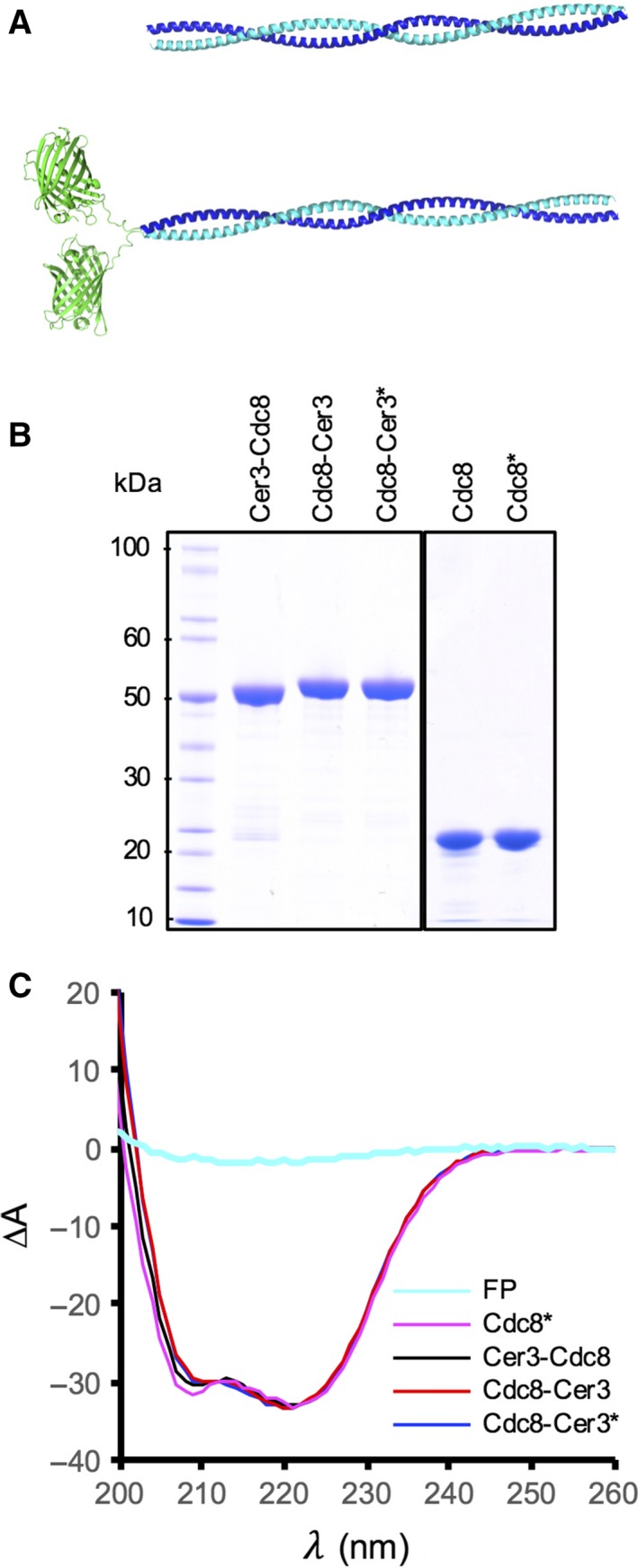
Tropomyosin proteins used in this study. (A) Predictive models of Tpm^Cdc8^ (upper panel) and Cerulean3‐Tpm^Cdc8^ (lower panel) dimers. (B) Coomassie blue‐stained SDS/PAGE analysis of purified wild‐type and Cerulean3‐tagged Tpm^Cdc8^ proteins. (C) CD Spectra of purified acetylated wt (magenta), amino‐terminally Cerulean3‐tagged (black), and both acetylated (blue) and unacetylated (red) carboxyl‐terminally Cerulean3‐tagged Tpm^Cdc8^ proteins. A CD spectrum of a fluorescent protein (cyan) is included for comparison. *Denotes amino‐terminally acetylated protein.

In order to determine the impact fusion of a fluorescent protein to the termini had upon the ability of tropomyosin to self‐polymerise, associate with actin and function within a cell, bacteria and fission yeast expression constructs were generated which allowed encoding of amino and carboxyl terminal fusions between the fission yeast tropomyosin, Tpm^Cdc8^, and the monomeric fluorescent protein, Cerulean3 28, each juxtaposed a poly‐glycine linker (Fig. 1A). The Cerulean3 fluorophore is a relatively bright, photo‐ and thermostable monomeric fluorescent protein, and due to its low emission wavelength, allows excellent spatial resolution in live cell imaging applications.

To facilitate biochemical analysis of the Tpm proteins, each Cerulean3 fusion (Cerulean3‐Tpm^Cdc8^ and Tpm^Cdc8^‐Cerulean3) together with the wild‐type protein was expressed and purified from *E. coli* cells (Fig. 1B). In addition, wild‐type and the Tpm^Cdc8^‐Cerulean3 carboxyl fusion which had been acetylated upon the amino‐methionine resides were isolated using a recombinant NatB acetylation system 25 (Fig. 1B – acetylated proteins denoted by *). The mass and purity of each protein was confirmed using mass spectroscopy analysis (Table 1).

**Table 1:** Summary of data presented

Protein	Expected Mass (kDa)	Determined mass (kDa)	*T*_m_ (°C)	Viscosity (cSt)	*K*_50%_ (μM)	Growth rate^a^ (∆OD_600_ h^−1^)	CAR ∅ constriction rate (μm·min^−1^)^a^	Cell length μm ($\overset{¯}{x}$ ± SD)^a^^,^ b	Myo52 movements^a^
Tpm^Cdc8^	18 964	18 963.9	33.4	1.08	2.7	–	–	–	–
*ace‐*Tpm^Cdc8^	19 006	19 006.4	35.4	1.22	0.6	0.08	0.088 ± 0.014	11.8 ± 3.0	+++
Cerulean3‐Tpm^Cdc8^	47 002	47 001.6	35.5	1.17	0.69	0.063	0.09 ± 0.007	13.9 ± 4.3	+
Tpm^Cdc8^‐Cerulean3	46 871	46 870.4	34.3	0.99	n.a.	–	0	24.2 ± 9.7	–
*ace*‐Tpm^Cdc8^‐Cerulean3	46 913	46 912.4	35.7	1.02	n.a.	0	0	–	–

a Determined in *cdc8.110* cells at 36 °C.

b GFP alone: 18.9 ± 4.3 μm.

**Figure 2:**
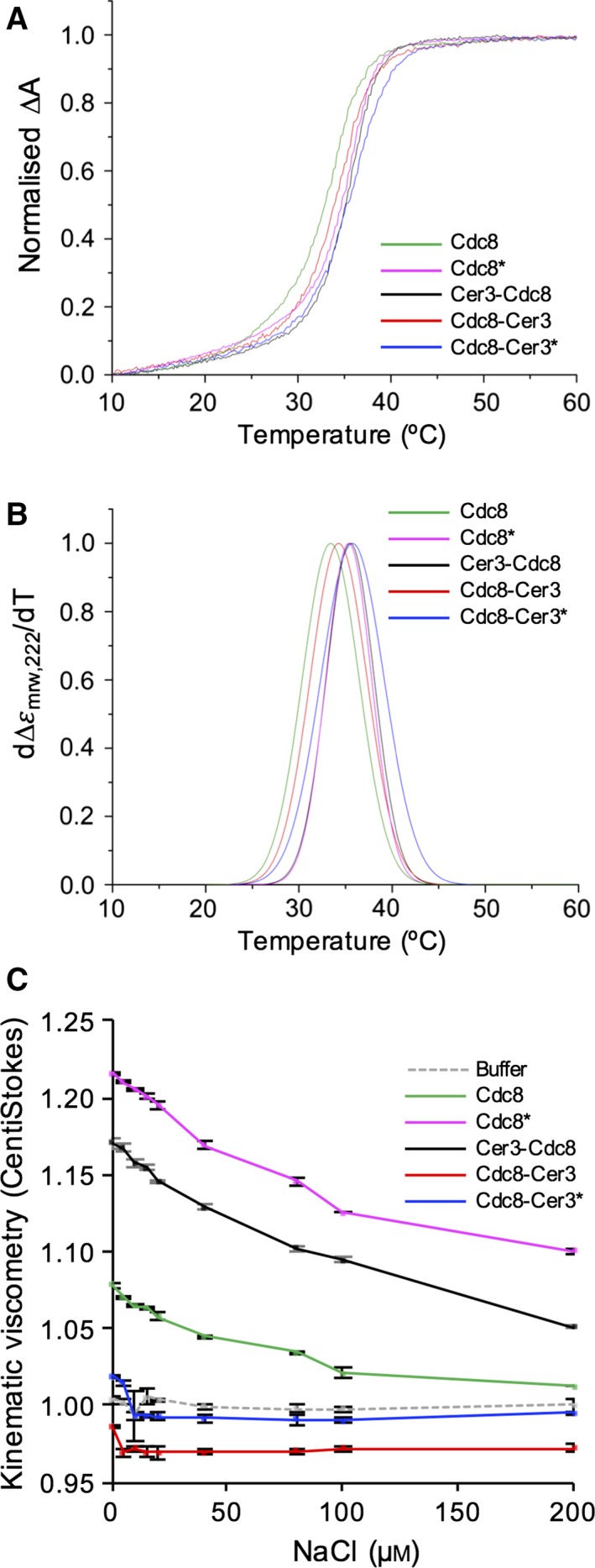
FP Fusions affect Tpm filament formation not themostability. (A) Normalised differential CD absorbance data for unacetylated Tpm^Cdc8^ (green), acetylated Tpm^Cdc8^ (magenta), Cerulean3–Tpm^Cdc8^ (black), aceteylated Tpm^Cdc8^–Cerulean3 (blue) and unaceylated Tpm^Cdc8^–Cerulean3 (red) at 222 nm. (B) First derivative plots at 222 nm of data described in (A). (C) Viscosity of 20 μm Tpm^Cdc8^ proteins and buffer (grey dashed) at increasing NaCl concentrations (0–200 mm) at 23 °C.

As a first step, Circular dichroism (CD) analyses were performed upon each protein to assess the impact the addition of Cerulean3 had on the thermal stability of the dimer. As reported previously 27, broad negative CD spectra peaks at 208 and 222 nm, consistent for α‐helical structures were observed for the wild‐type and modified forms of the Tpm^Cdc8^ protein (Fig. 1C). The presence of the Cerulean3 fusion had no significant impact upon the overall CD spectra, as the relative CD signal of an equivalent amount of FP alone is 5% that of that observed for the dimeric coiled‐coil Tpm^Cdc8^ proteins (Fig. 1C). Normalised melting curves for absorbance at 222 nm of each protein are shown in Fig. 2A. Once each melting curve was acquired, the protein was cooled and subjected to two further runs. CD spectra were acquired at the start of each run, and were identical for each protein (not shown), indicating each Tpm^Cdc8^ was able to rapidly refold to its original state. Figure 2B illustrates the first derivative plots for these data, from which mid‐point melting temperatures (*T*_m_) were calculated (Table 1). The *T*_m_ of unmodified and amino‐terminally acetylated Tpm^Cdc8^ were equivalent to values established previously 27. The addition of Cerulean3 to the carboxyl end of Tpm^Cdc8^ had a minimal effect on the thermostabiltiy of Tpm^Cdc8^, increasing the *T*_m_ from 33.4 °C to 34.3 °C. However, fusing Cerulean3 to the amino‐terminal of the Tpm^Cdc8^ increased the thermal stability of the protein to equal that of acetylated wild‐type Tpm^Cdc8^, 35.5 °C. Similarly, acetylating the amino‐terminal methionine of Tpm^Cdc8^‐Cerulean3 increased the *T*_m_ to an equivalent value (*T*_m_ = 35.7 °C). These data confirm that the addition of the Cerulean3 polypeptide to either end of Tpm^Cdc8^ had no significant detrimental effect upon stability of the Tpm^Cdc8^ coiled‐coil protein.

The additional mass from Cerulean3 at the termini of Tpm^Cdc8^ has the potential to impact the ability to form end‐to‐end interactions between the amino and carboxyl termini of Tpm^Cdc8^ dimers and therefore allow the protein to polymerise. Viscometry was used to investigate the effect the addition of the fluorescent protein had upon the ability of Tpm^Cdc8^ to form filaments. In this assay, a higher viscosity correlates with a greater capacity to form filaments. Upon the addition of NaCl the strength of ionic interactions between the ends of Tpm^Cdc8^ proteins was reduced, which is reflected in a reduction in viscosity. Figure 2C shows viscosity data for each Tpm^Cdc8^ and Tpm^Cdc8^ fluorescent protein fusion. The viscosity of each protein in the absence of NaCl is shown in Table 1. Acetylation increases the propensity of Tpm^Cdc8^ to form filaments, which was reflected in an increase in the viscosity from 1.08 centiStokes (cSt) for the unmodified protein, to 1.22 cSt for the amino‐terminally modified form. The viscosity data for tagged Tpm^Cdc8^ indicates the end‐to‐end interactions are affected by the addition of the Cerulean3 fluorophore to either terminus of the protein. In the absence of salt, amino‐terminally tagged Tpm^Cdc8^ formed end‐to‐end interactions and gave a viscosity reading of 1.17 cSt, which is lower than the wild‐type acetylated protein. The carboxyl terminal fusion had a more significant impact upon Tpm^Cdc8^ filament formation, and abolished the ability of the protein to form end‐to‐end interactions, as the viscosity values for the unacetylated and acetylated Tpm^Cdc8^‐Cerulean3 proteins of 0.99 and 1.02 cSt, respectively, are equivalent to buffer only control samples. These data indicate that fusion of the Cerulean3 fluorescent protein to either end of Tpm^Cdc8^ impacts the end‐to‐end interactions between adjacent tropomyosin dimers.

**Figure 3:**
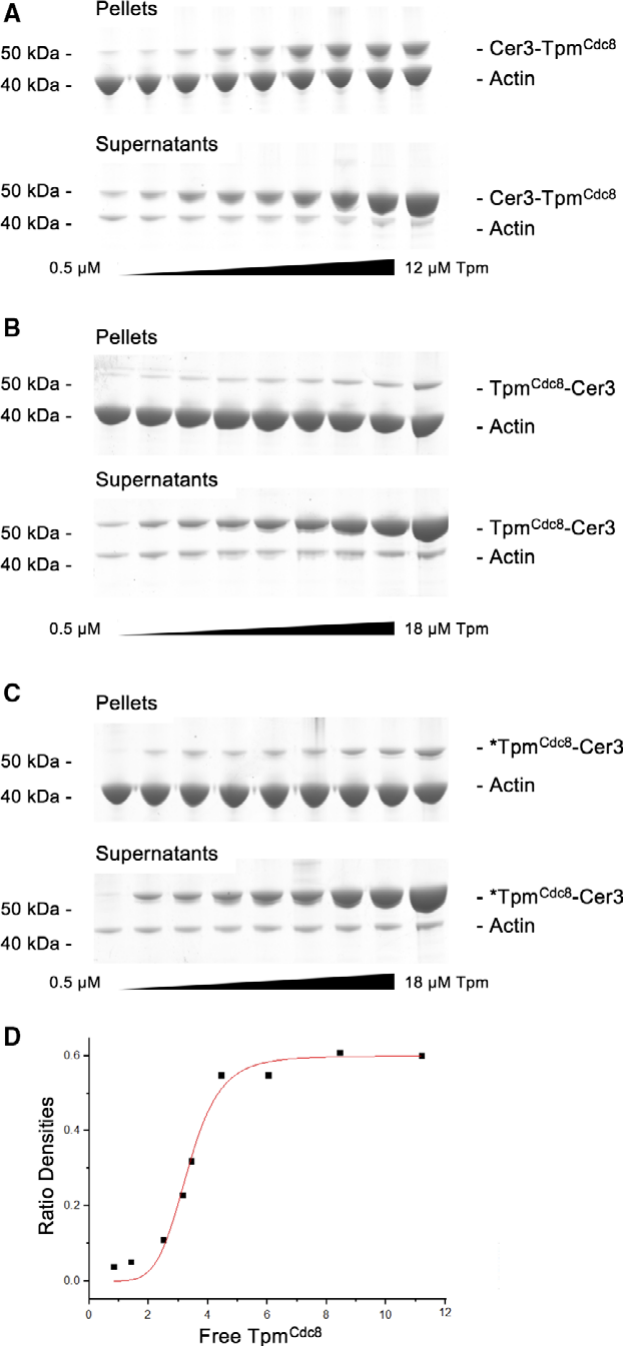
Actin‐binding assays of FP Tpm fusions. SDS/PAGE gels of pellet and supernatant fractions from cosedimentation assays of (A) Cerulean3‐Tpm^Cdc8^, (B) Tpm^Cdc8^‐Cerulean3 and (C) Nt‐acetylated Tpm^Cdc8^‐Cerulean3. (D) Binding curve of the free Tpm^Cdc8^ concentration against the ratio of density of actin, for Cerulean3‐Tpm^Cdc8^, measured by densitometry of cosedimentation SDS/PAGE gels. Curves represent Hill equation lines of best fit.

In addition to effects on Cdc8 stability and end‐to‐end contacts, we performed cosedimentation experiments to determine whether the addition of the Cerulean3 fusion had an impact upon the ability of Tpm^Cdc8^ interactions with actin. Examples of Coomassie‐stained SDS/PAGE gels used to determine the binding affinity of Cerulean3‐Tpm^Cdc8^ and both unmodified and amino‐terminally acetylated Tpm^Cdc8^‐Cerulean3 are shown in Fig. 3. In each experiment shown the lower faster migrating bands correspond to actin, with the density remaining approximately constant between each sample. The upper bands are Tpm^Cdc8^ Cerulean3 fusions and the density increases as dimer concentrations increase from left to right. Figure 3A shows a typical example of a binding experiment for the amino‐terminal Cerulean3‐Tpm^Cdc8^ fusion. This protein can be seen to bind strongly to actin as a faint band can be observed in the pellet with as little as 0.5 μm Cerulean3‐Tpm^Cdc8^, with a much denser band visible at 12 μm Tpm. A typical binding curve from a Cerulean3‐Tpm^Cdc8^‐binding experiment is shown in Fig. 3D. The *K*_50%_ for this fusion was calculated to be 0.69 μm, which is similar to the previously reported *K*_50%_ value for acetylated Tpm^Cdc8^ (0.46 μm) 8, 27. In contrast, binding data for both unacetylated (Fig. 3B) and acetylated (Fig. 3C) Tpm^Cdc8^‐Cerulean3 illustrate both forms of the carboxyl terminal fusion bind weakly to actin. Even at 20 μm Tpm, only a small proportion of the protein is in the pellet fraction, while a large amount of Tpm remains in the supernatant. Both acetylated and unacetylated forms of Tpm^Cdc8^‐Cerulean3 bound extremely weakly to actin and binding curves could not be generated, as the binding coefficients were estimated to be greater than 20 μm.

In conclusion, *in vitro* analyses show that the addition of a fluorescent protein fusion to either end of the Tpm^Cdc8^ did not impact the α‐helical content or thermal stability of the protein. However, carboxyl terminal fusions inhibited both the ability of Tpm to form end‐to‐end contacts, and ability to associate with actin. In contrast, the amino‐terminal Cerulean3‐Tpm^Cdc8^ fusion protein has properties that closely mimic the acetylated endogenous protein.

**Figure 4:**
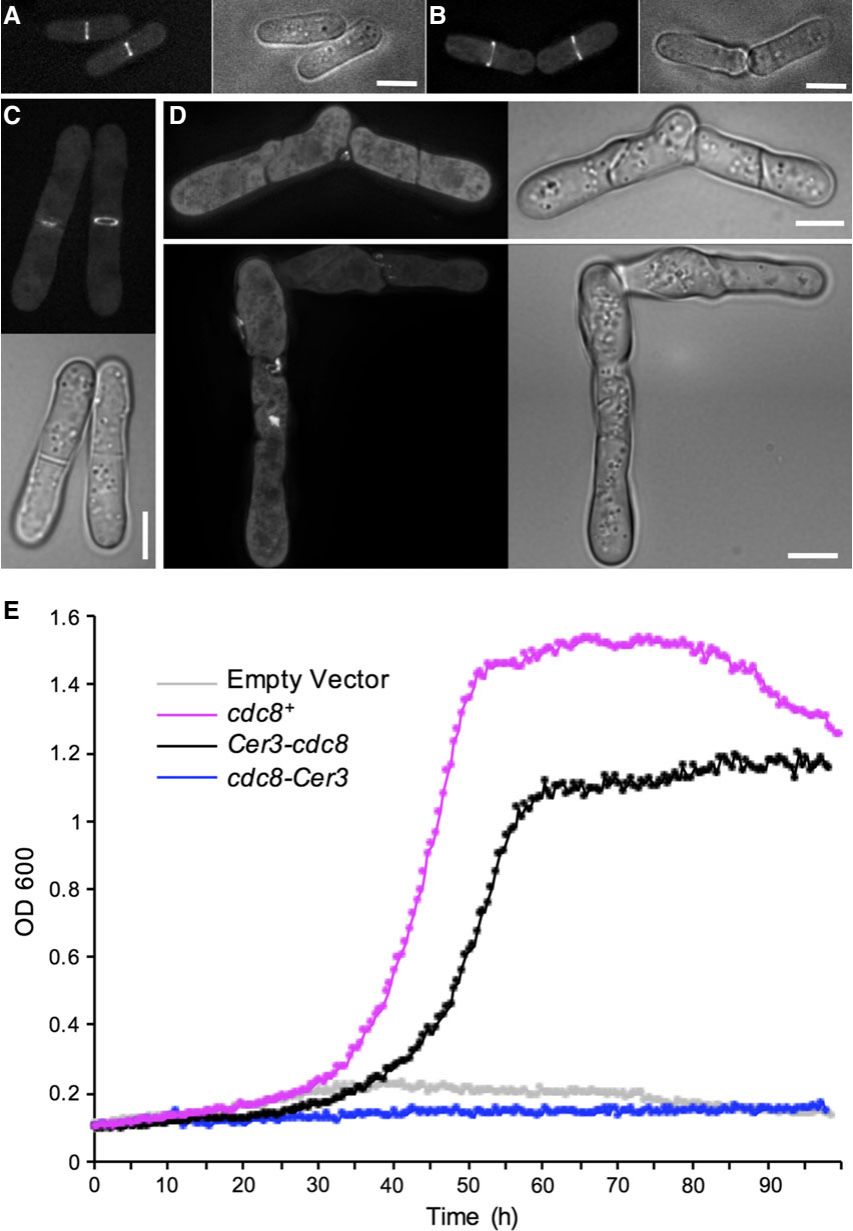
Ability of FP Tpm fusions to associate with and support CAR function. Localisation of Cerulean‐Tpm^Cdc8^ (A and C) and Tpm^Cdc8^‐Cerulean3 (B and D) in wild‐type (A and B) and *cdc8.110* (C and D) cells at at 36 °C. (E) Growth curves of *cdc8.110* cells containing plasmids encoding for wild‐type *Tpm* ^*cdc8+*^ (magenta), *Cerulean3‐Tpm* ^*cdc8*^ (black), *Tpm* ^*cdc8*^ *‐Cerulean3* (blue) or an empty vector (grey) cultured at 36 °C. Scale bars: 5 μm.

We next explored the ability of each fusion to localise and complement the function of the endogenous Tpm^Cdc8^ protein within the fission yeast cell. Having previously established that expression levels of GFP‐Tpm^Cdc8^ from the *nmt41* promoter reflect Tpm^Cdc8^ levels from the endogenous locus 8, we chose to express the Tpm^Cdc8^ fusions using the same repressible promoter 23. We first examined the localisation of the Cerulean3‐Tpm^Cdc8^ and Tpm^Cdc8^‐Cerulean3 fusion proteins in wild‐type cells. Each protein was seen to associate with the actin polymers within the contractile CAR, when endogenous Tpm^Cdc8^ was present in the cell (Fig. 4A,B). In contrast, only the Cerulean3‐Tpm^Cdc8^ was seen to localise to and support the formation of the actin ring in cells possessing the temperature‐sensitive *cdc8‐110* allele when held at the restrictive temperature (Fig. 4C, D) Consistent with these findings comparison of growth kinetics between *cdc8‐110* cells containing plasmid born copies of wild‐type *Tpm* ^*cdc8*^, *Tpm* ^*cdc8*^ *‐Cerulean3* or *Cerulean3‐Tpm* ^*cdc8*^ showed that only the amino‐terminal fusion permitted growth, at a rate approaching that of wild‐type at the restrictive temperature of 36 °C (Fig. 4E, Table 1). However, while Cerulean3‐Tpm^Cdc8^ facilitated the formation of an actin ring, cells were significantly longer than wild‐type (Fig. 4C, Table 1) demonstrating the normal timing of cell division was disrupted. These data suggest that the fusions may be able to form heterodimers with endogenous unlabelled tropomyosin, as only the amino‐terminal Cerulean3‐Tpm^Cdc8^ fusion could form dimers with the capacity to form filaments and associate with actin in the absence of the wild‐type protein. This is consistent with the *in vitro* analysis (Figs 2 and 3).

**Figure 5:**
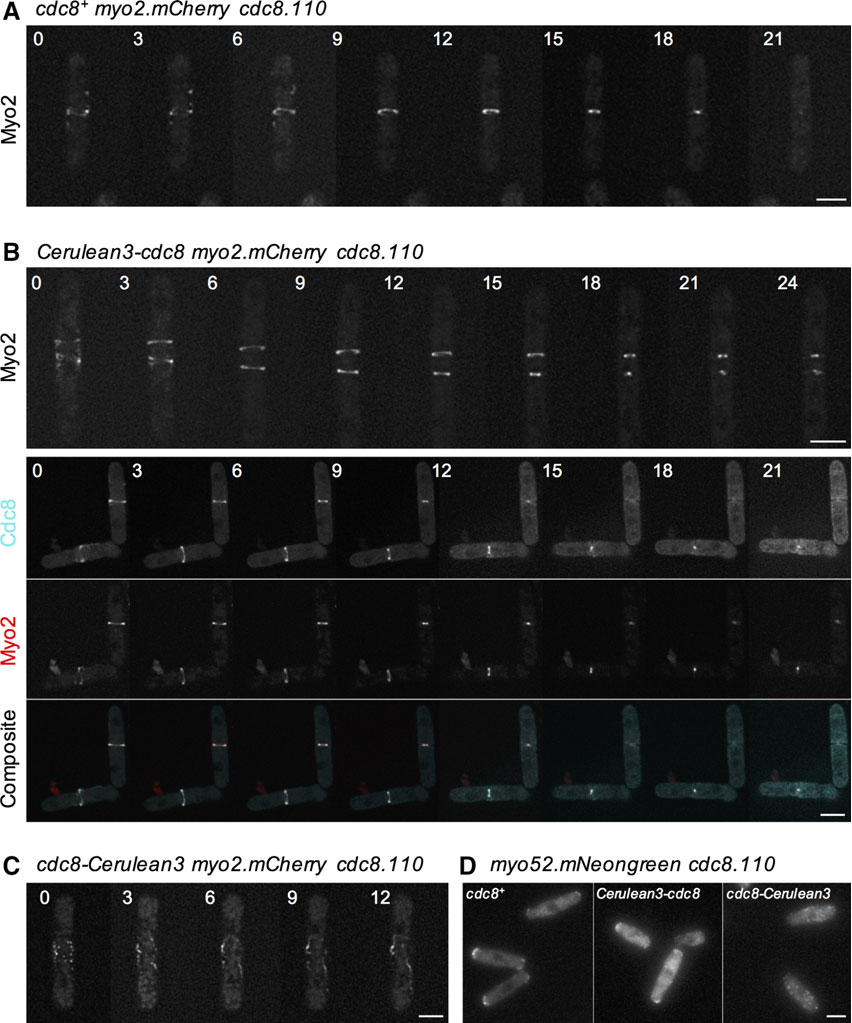
Myosin regulatory function of FP Tpm fusions. Montages of timelapse frames of (A) *cdc8.110 myo2*.*mCherry* pREP41*Tpm* ^*cdc8+*^, (B) *cdc8.110 myo2*.*mCherry* pREP41Cerulean3‐*Tpm* ^*cdc8*^, and (C) *cdc8.110 myo2*.*mCherry* pREP41*Tpm* ^*cdc8*^‐Cerulean3 cells grown at 36 °C. Images show mCherry (A–C) and Cerulean (B‐lower panels) signals Timings are shown in minutes. (D) Micrographs of mNeongreen signal from *cdc8.110 myo52*.*mNeongreen* cells expressing different *Tpm* ^*cdc8*^ constructs. Scale bars: 5 μm.

As Tpm^Cdc8^ plays a key role in regulating the motor activity of both class II and V myosins 10, 11, 27, we next determined how the fusions affected the ability of the coiled‐coil protein to regulate myosin motor activity *in vivo*. Myo2 is the only essential myosin within the *S. pombe* cell, and is required for the assembly of the CAR from actomyosin containing medial nodes 29, 30, as well as providing the contractile force required for this organelle's constriction 31, 32, 33. As a readout on the ability of each fusion protein to regulate the contractile activity of myosin II 8, 15, 34, we measured the rate on CAR constriction in *cdc8.110 myo2.mCherry* cells at 36 °C. Consistent with our previous observation that the *myo2.mCherry* allele is functional at this temperature 9, these cells formed normal contractile rings which constricted at an average rate of 0.088 ± 0.014 μm·min^−1^ (mean ∆CAR‐diameter rate ± SD), when wild‐type Tpm^Cdc8^ was expressed from an episomal plasmid (Fig. 5A). In comparable cells expressing Cerulean3‐Tpm^Cdc8^, the CAR constricted at a rate of 0.09 ± 0.007 μm·min^−1^. However, in more than 30% of these mitotic *cdc8.110 myo2.mCherry* Cerulean3‐*Tpm* ^*cdc8*^ cells, we observed MyoII incorporated into multiple contractile actomyosin rings (Fig. 5B). Thus, expression of Cerulean3‐Tpm^Cdc8^ disrupted the regulation of node‐dependent formation of the CAR, but once formed the actin‐Cerulean3‐Tpm^Cdc8^ copolymers were capable of regulating normal myosin II‐dependent contraction. In contrast, *cdc8.110 myo2.mCherry* cells expressing Tpm^Cdc8^‐Cerulean3 failed to form a stable CAR when cultured at 36 °C. Instead Myo2 foci concentrated to the cell equator and incorporated into randomly organised contractile filaments or aggregates (Fig. 5C). Thus, unable to form a functional CAR these cells failed to complete cytokinesis, leading to a loss of viability (Fig. 5C).

Finally, we explored the impact tagging Tpm^Cdc8^ had upon the ability of the protein to regulate myosin V function. The fission yeast myosin V, Myo52, associates with interphase actin‐Tpm^Cdc8^ copolymers, and moves towards the barbed end of these filaments to deliver cellular cargoes to the growing tips of the cell 35, 36, 37. In yeasts Tpm plays a critical role in modulating the duty‐ratio of this dimeric motor protein to promote processive movement upon actin 11, 38. Using a *cdc8.110 myo52.mNeogreen* strain, we tested the ability of each Tpm^Cdc8^ fusion to facilitate the actin‐dependent movement of the myosin V protein. Myo52 fails to accumulate at the growing ends of fission yeast cells lacking functional Tpm^Cdc8^ 39. Consistent with an inability to associate with formin‐nucleated tropomyosin associated actin polymers, Myo52 movements were not observed in *cdc8.110 myo52.mNeogreen* cells expressing Tpm^Cdc8^‐Cerulean3 and the myosin V was not seen to accumulate at ends of cells (Fig. 5D), but instead localise to randomly dispersed foci throughout the cell. In contrast, Myo52 was seen at the ends of cells expressing wild‐type Tpm^Cdc8^ or the amino‐terminal Cerulean3‐Tpm^Cdc8^ fusion. However, in comparison to Tpm^Cdc8^‐containing cells, fewer Myo52 movements were observed within the cytosol in the presence of Cerulean3‐Tpm^Cdc8^ (not shown). Thus, while this fusion is capable of forming interphase actin polymers, it does not promote normal processive movement of myosin V within the cell.

## Discussion

Over the last decade tropomyosin–fluorescent protein fusions have been used to study the organisation of actin polymers within diverse cell types 17, 18, 19, 20, 21. In each of these studies either the wild‐type protein is present within the cell or the Tpm being labelled is nonessential, thus the ability of the fusion‐protein to complement its function has not been tested. Given the terminal regions of a Tpm are critical for modulating its ability to (a) form end‐to‐end contacts; (b) regulate cooperative movements on the actin filament; (c) interact with regulatory proteins; and (d) interact with specific actin polymers at precise cellular locations, we decided to investigate the impact FP‐Tpm fusions had upon the biophysical properties and function of this essential cytoskeletal protein. Localisation studies of globular proteins have used tagging strategies where an FP is inserted at a surface loop within the protein sequence, resulting in fusion proteins with varying degrees of functionality 40, 41, 42. However, this strategy is not suitable for use with tropomyosins as they are single alpha‐helices, and insertion of an FP with associated flexible linkers would not only disrupt its packing on the actin surface but would also change the flexibility and shape of the tropomyosin filament, and thereby disrupt its ability to regulate actin function 43.

Having a single Tpm protein, Tpm^Cdc8^, makes the fission yeast an attractive model for studying the regulation and function of this conserved dimeric protein. We show that fusing Cerulean3 to the carboxyl terminus of this Tpm disrupts the ability of this protein to form end‐to‐end contacts and abolishes its ability to interact with actin and function within a cell, even when amino‐terminally acetylated. In contrast, the addition of the monomeric FP to the amino‐terminal of Tpm^Cdc8^ confers the resultant protein with physical properties equivalent to the acetylated wild‐type protein. This is consistent with previous studies on vertebrate tropomyosins which indicate amino‐terminal fusions do not interfere with head‐to‐tail self‐association and actin‐binding properties of tropomyosin 19, 44, 45. However, modifications to the amino‐terminus of the Tpm protein not only affect interactions with other actin‐binding proteins 9, 45 but also change the structure at the end of actin‐Tpm copolymers.

We have previously shown the cellular distribution of Tpm^Cdc8^ and its interactions with actin and myosin are modulated by amino‐terminal acetylation 9, 27, a modification common to all Tpms studied to date 4. This is consistent with the findings presented here. While Cerulean3‐Tpm^Cdc8^ binds to actin and regulates myosin II activity during CAR constriction to the same extent as wild‐type protein, its expression often disrupts initial polymerisation events and results in the disruption of normal actin ring formation and a subsequent delay in cell division. This supports models where the Tpm amino‐terminus is crucial during actin‐Tpm cofilament nucleation and growth. Thus, the addition of FP tags to the end of the Tpm changes the landscape at the end of the actin polymer, disrupting normal amino‐terminal signalling. Consistent with this idea, a single amino‐acid substitution within the amino‐terminus of Tpm^Cdc8^ (E6K) has been shown to biophysically mimic the effect of amino‐terminal acetylation, however, unlike an amino‐terminal fusion, can fully complement endogenous *cdc8* function 8, 27. The terminus of the Tpm^Cdc8–E6K^ is almost identical to the wild‐type protein, and is acetylated *in vivo*, again supporting the hypothesis that the biophysical landscape at the amino‐terminus of the Tpm^Cdc8^ protein is crucial for its proper regulation and function.

Both Tpm^Cdc8^ fusion are capable of localising to the CAR in cells possessing the wild‐type *cdc8* ^*+*^ allele. However, only Cerulean3‐Tpm^Cdc8^ was able to localise and support growth in the absence of functional endogenous protein. This indicates the carboxyl‐terminal fusion, which is unable to form end‐to‐end polymers or associate with actin alone, is capable of forming heterodimers with endogenous Tpm protein. Similarly, GFP fusions with budding yeast Tpm1 localises to the *S. pombe* CAR, but do not complement Tpm^Cdc8^ function. Thus, the ability of a FP‐Tpm fusion to decorate actin polymers in a cell does not provide any indication of its functionality at any level.

In conclusion, while the impact of fusing a fluorescent protein to the terminus of a Tpm is likely to vary between different Tpm isoforms and cell types, it is clear from data presented here that their functionality and biophysical properties should be determined and shown to reflect that of the wild‐type protein. Unless this is the case, care should be taken in drawing conclusions from data generated from using them.

## Author contributions

HRB performed the experiments; HRB, MAG and DPM conceived and designed the experiments; HRB, MAG and DPM analyzed the data; DPM wrote the manuscript.
